# Supplementary material for: Data-based analysis, modelling and forecasting of the COVID-19 outbreak
Source: PLoS One. 2020 Mar 31;15(3):e0230405. doi: 10.1371/journal.pone.0230405 (PMC7108749; doi:10.1371/journal.pone.0230405)
Supplement: S1 Table — (PDF) [file pone.0230405.s001.pdf]

Data-Based Analysis, Modelling and Forecasting of the COVID-19 outbreak

Cleo Anastassopoulou[1*], Lucia Russo[2], Athanasios Tsakris[1], Constantinos Siettos[3*]

1 Department of Microbiology, Medical School, University of Athens, Athens, Greece
2 Consiglio Nazionale delle Ricerche, Science and Technology for Energy and Sustainable Mobility, Napoli, Italy
3 Dipartimento di Matematica e Applicazioni "Renato Caccioppoli", Università degli Studi di Napoli Federico II, Napoli, Italy

S1 Table. Reported cumulative numbers of cases for the Hubei region, China for the period January 11-February 10

Date	Infected	Deaths	Recovered
11	41	1	2
12	41	1	6
13	41	1	7
14	41	1	7
15	41	2	12
16	45	3	12
17	62	3	16
18	121	3	21
19	198	4	25
20	270	6	25
21	375	9	25
22	444	17	28
23	549	24	31
24	729	39	34
25	1052	52	44
26	1423	76	46
27	2714	100	49
28	3554	125	82
29	4586	162	92
30	5806	204	118
31	7153	249	168
01	9074	294	218
02	11177	350	295
03	13522	414	397
04	16678	479	522
05	19665	549	633
06	22112	618	817
07	24953	699	1218
08	27100	780	1480
09	29631	871	1795
10	31728	974	2222
